# Supplementary material for: MetaRibo-Seq measures translation in microbiomes
Source: Nat Commun. 2020 Jun 29;11:3268. doi: 10.1038/s41467-020-17081-z (PMC7324362; doi:10.1038/s41467-020-17081-z)
Supplement: Supplementary file 10 — Supplementary Data 7 [file 41467_2020_17081_MOESM10_ESM.zip › File2/Confidence_High_Taxonomy/182695_out.krona.html]

Javascript must be enabled to view this page.

members
magnitude
magnitudeUnassigned
count
unassigned
taxon
rank

182695\_out

2

2
2
superkingdom

phylum
2
976

class
2
200643

171549
2
order

815
2
family

2
genus
816


SRS019787\_contig\_number\_5039
1
species
329854


SRS016267\_contig\_number\_1911
species
1
357276
